# Strain Kaplan of Pseudorabies Virus Genome Sequenced by PacBio Single-Molecule Real-Time Sequencing Technology

**DOI:** 10.1128/genomeA.00628-14

**Published:** 2014-07-17

**Authors:** Dóra Tombácz, Donald Sharon, Péter Oláh, Zsolt Csabai, Michael Snyder, Zsolt Boldogkői

**Affiliations:** a Department of Medical Biology, Faculty of Medicine, University of Szeged, Szeged, Hungary; b Department of Genetics, School of Medicine, Stanford University, Stanford, California, USA

## Abstract

Pseudorabies virus (PRV) is a neurotropic herpesvirus that causes Aujeszky’s disease in pigs. PRV strains are widely used as transsynaptic tracers for mapping neural circuits. We present here the complete and fully annotated genome sequence of strain Kaplan of PRV, determined by Pacific Biosciences RSII long-read sequencing technology.

## GENOME ANNOUNCEMENT

Pseudorabies virus (PRV), also known as Aujeszky’s disease virus or suid herpesvirus 1, a member of the *Alphaherpesvirinae* subfamily, causes significant abortion and morbidity in pigs, the natural host of the virus (1). PRV is a useful model organism for studies of the pathogenesis of herpesviruses. The genetically modified strains are powerful tracers for mapping neuronal circuits (2–6), are tools in gene and cancer therapy (7), and serve as viral vectors for gene delivery into mammalian neurons (3, 4) and cardiomyocytes (8); PRVs have also been employed as live vaccines against Aujeszky’s disease (9–11). Further, attenuated vaccine strains of PRV are valuable models for novel vaccine development against varicella-zoster virus (VZV) and herpes simplex virus 1 and 2 (HSV-1 and HSV-2, respectively) (12).

The currently available genome sequences of PRV contain several discrepancies, mainly in intergenic repetitive regions (GenBank accession no. JF797218.1), and the totally annotated version of genome sequence is a composite of six different PRV strains (GenBank accession no. NC_006151.1). We have sequenced the PRV Kaplan genome with Pacific Biosciences single-molecule long-read sequencing technology (Pacific Biosciences, Menlo Park, CA, USA) in order to upgrade the draft sequences, reconstruct the GC-rich and repetitive regions of the genome, and extract epigenetic information. The availability of the completely annotated genome and the single-base resolution methylation map of strain Kaplan will aid in understanding the control of viral gene expression at different levels. Investigations of the PRV genome and gene functions are expected to result in the development of effective vaccines and direct practical applications in gene, cancer, and antiviral therapies.

Sequencing of purified virion DNA was carried out on the Pacific Biosciences RSII sequencer. SMRTbell template libraries were prepared from the DNA, as previously described (13, 14), using standard protocols for 6-kb and 20-kb library preparation. Sequencing was performed in five single-molecule real-time (SMRT) cells with P5 DNA polymerase and C3 chemistry (P5-C3) yielding a total of 78,111 reads and an extremely high coverage (1,200×) throughout the genome.

The sequencing reads were processed and mapped to the respective reference sequences with the BLASR mapper (https://github.com/PacificBiosciences/blasr) and the Pacific Biosciences SMRT Analysis pipeline (https://github.com/PacificBiosciences/SMRT-Analysis/wiki/SMRT-Pipe-Reference-Guide-v2.0) using the standard mapping protocol.

The protein-coding genes were predicted by GATU (15). Manual annotation was used to identify other genomic features. Annotation of a previously unknown noncoding RNA (named Close to OriL [CTO]), a newly discovered splice site of the early protein 0 gene, and new isoforms of 11 protein-coding genes are based on RNAseq data (our unpublished data). MicroRNA (miRNA) annotation was based on the precursor miRNAs found in strains NIA-3 and Ea.

The complete genome of strain Kaplan of PRV is characterized as a double-stranded linear DNA composed of 143,423 bp, with an average G+C content of 73.59%. PRV contains 70 protein-coding genes (11 genes have different isoforms), two latency-associated transcripts, and a long noncoding RNA, and its genome predicts 16 miRNAs.

### Nucleotide sequence accession number.

The complete genome of strain Kaplan of pseudorabies virus was assigned DDBJ/EMBL/GenBank accession no. KJ717942.
